# Altered Time Awareness in Dementia

**DOI:** 10.3389/fneur.2020.00291

**Published:** 2020-04-21

**Authors:** Maï-Carmen Requena-Komuro, Charles R. Marshall, Rebecca L. Bond, Lucy L. Russell, Caroline Greaves, Katrina M. Moore, Jennifer L. Agustus, Elia Benhamou, Harri Sivasathiaseelan, Chris J. D. Hardy, Jonathan D. Rohrer, Jason D. Warren

**Affiliations:** ^1^Dementia Research Centre, UCL Queen Square Institute of Neurology, University College London, London, United Kingdom; ^2^Preventive Neurology Unit, Wolfson Institute of Preventive Medicine, Queen Mary University of London, London, United Kingdom

**Keywords:** time perception, clockwatching, Alzheimer's disease, frontotemporal dementia, primary progressive aphasia, semantic dementia, voxel-based morphometry

## Abstract

Our awareness of time, specifically of longer intervals spanning hours, days, months, and years, is critical for ensuring our sense of self-continuity. Disrupted time awareness over such intervals is a clinical feature in a number of frontotemporal dementia syndromes and Alzheimer's disease, but has not been studied and compared systematically in these diseases. We used a semi-structured caregiver survey to capture time-related behavioral alterations in 71 patients representing all major sporadic and genetic syndromes of frontotemporal dementia, in comparison to 28 patients with typical Alzheimer's disease and nine with logopenic aphasia, and 32 healthy older individuals. Survey items pertained to apparent difficulties ordering past personal events or estimating time intervals between events, temporal rigidity and clockwatching, and propensity to relive past events. We used a logistic regression model including diagnosis, age, gender, and disease severity as regressors to compare the proportions of individuals exhibiting each temporal awareness symptom between diagnostic groups. Gray matter associations of altered time awareness were assessed using voxel-based morphometry. All patient groups were significantly more prone to exhibit temporal awareness symptoms than healthy older individuals. Clinical syndromic signatures were identified. While patients with typical and logopenic Alzheimer's disease most frequently exhibited disturbed event ordering or interval estimation, patients with semantic dementia were most prone to temporal rigidity and clockwatching and those with behavioral variant frontotemporal dementia commonly exhibited all these temporal symptoms as well as a propensity to relive past events. On voxel-based morphometry, the tendency to relive past events was associated with relative preservation of a distributed left-sided temporo-parietal gray matter network including hippocampus. These findings reveal a rich and complex picture of disturbed temporal awareness in major dementia syndromes, with stratification of frontotemporal dementia syndromes from Alzheimer's disease. This is the first study to assess symptoms of altered temporal awareness across frontotemporal dementia syndromes and provides a motivation for future work directed to the development of validated clinical questionnaires, analysis of underlying neurobiological mechanisms and design of interventions.

## Introduction

Our capacity to experience and calibrate the passage of time anchors us in the flux of sensory experience and allows us to track external events, conduct our daily affairs and most fundamentally, maintain a sense of self-continuity from past to future (1–3). Our awareness of time is not dictated simply by the clock: it is a complex and elastic, subjective psychological construct, encompassing multiple, hierarchically embedded scales, ranging from fractions of a second to an entire lifetime (4–6). Brief time intervals are more directly accessible to laboratory analysis and accordingly, most neuropsychological data on time perception and awareness relate to shorter timescales (7–9). However, many of our psychologically salient experiences unfold over longer timespans that are challenging to study experimentally.

Distributed cortical and subcortical brain networks—including prefrontal and insular cortices (2, 5, 10–14), parietal cortex (15–17), hippocampus (3, 18–20), and basal ganglia (21–23)—have been associated with temporal encoding at different time scales and mediating different kinds of temporal computations (4, 6, 24, 25). Normal temporal awareness across timescales and in particular, integration of external clock time with internal bodily or “subjective” time is likely to depend on interactions between large-scale neural networks: for example, fronto-striatal time-keeping circuitry (26–29) operating in concert with the so-called “default mode” temporo-parietal network that mediates self-awareness and self-projection (12, 25, 30).

This neural network paradigm provides a rationale for anticipating and understanding alterations of subjective temporal awareness accompanying neurodegenerative pathologies. These pathologies characteristically and selectively target the distributed neural networks implicated in temporal processing in the healthy brain (31–35). It follows that neurodegenerative disorders are likely to have overlapping but separable phenotypes of abnormal temporal awareness, arising from the profiles of network involvement they produce. From a neurobiological perspective, better definition of temporal processing mechanisms in these diseases might provide novel insights into their pathophysiology. However, the clinical features and brain substrates of altered temporal awareness in major dementias such as Alzheimer's disease (AD) and in particular, frontotemporal dementia (FTD), have not been systematically assessed and compared.

Several questionnaires and interview protocols have been developed to look at subjective time awareness in both healthy and clinical populations (36–39). Notably, the Autobiographical Interview (40) has been useful in demonstrating impaired retrieval of past autobiographical memories (41, 42), and diminished ability to project into the future (43) in patients diagnosed with AD. Distortions of subjective time estimation (44–46), confusion about temporal ordering and reduced self-projection in time (46–49) have also been described in AD. Deficits of temporal processing in AD are not simply due to deteriorating episodic memory for the details of events, but rather the sequencing of those events in relation to one another, suggesting a more fundamental disorder of the mental timeline.

Temporal processing abnormalities have also been observed in FTD. This is a clinically and pathologically heterogeneous group of diseases (31), comprising three canonical clinico-anatomical syndromes: behavioral variant FTD (bvFTD; led by impaired socio-emotional signal processing and reactivity, with atrophy predominantly of prefrontal and insular cortices and their connections) and the language-led syndromes of semantic dementia (SD; led by degradation of vocabulary and conceptual knowledge with selective left anterior temporal lobe atrophy) and progressive non-fluent aphasia (PNFA; led by speech and language output failure with predominant left-sided peri-Sylvian atrophy). Impaired perception and generation of temporal intervals and patterns over short time intervals attributed to a deranged internal “clock” mechanism have been described in bvFTD and PNFA (50, 51). Over longer timescales, difficulties with prospection and retrospection have been documented in bvFTD and SD (49, 52–56). There are currently no validated instruments to assess clockwatching and more general inflexibility or obsessionality around time, although these are frequently associated with both bvFTD and SD (31, 57, 58). Impaired awareness of time and associated disruptions of socio-emotional behaviors in these diseases potentially take a substantial toll on patient well-being and care burden and therefore constitute a significant clinical issue.

Here we addressed the issue of temporal awareness in FTD in a cohort of patients representing all canonical syndromes of FTD (bvFTD, SD, and PNFA), in relation to healthy older individuals and a cohort of patients with typical amnestic AD and its major, language-led variant phenotype, logopenic aphasia (LPA). We surveyed temporal behavioral alterations which we hypothesized on clinical grounds to be particularly pertinent to the target syndromes and to the timeframes of daily life and autobiographical experience. We did not set out in this first study to characterize temporal symptoms in detail. Rather, our principal objective here was to survey the kinds of altered temporal awareness that occur in FTD, to estimate the relative proportions of patients with different FTD syndromes exhibiting symptoms of altered temporal awareness and to explore possible differences with respect to typical and amnestic AD. We assessed structural neuroanatomical correlates of altered temporal awareness using voxel-based morphometry (VBM) of patients' brain magnetic resonance images.

Based on clinical observations, we hypothesized that patients with AD syndromes (both the typical amnestic and the language-led phenotypes) would exhibit particularly prominent disturbances of temporal interval estimation and event ordering. In contrast, we hypothesized that patients with FTD syndromes (especially bvFTD and SD) would particularly exhibit reduced temporal flexibility and clockwatching. Based on available neuroimaging evidence in the healthy brain (2, 4, 5, 11, 13, 15–17, 20, 25, 28, 59–61), we further hypothesized that alterations of temporal awareness would have neural network correlates, reflecting the relative degree of involvement of posterior temporo-parietal cortices and hippocampus (engaged in temporal sequencing) vs. prefrontal and antero-mesial temporal cortices (engaged in temporal scheduling, appraisal, and valuation).

## Methods

### Participants

Seventy-one patients with FTD (34 bvFTD, 17 SD, 20 PNFA), twenty-eight patients with a typical memory-led syndrome of AD (hereafter, AD) and nine patients with LPA were recruited via a specialist cognitive disorders clinic. Thirty-two age-matched healthy individuals with no history of neurological or active psychiatric illness were recruited via the departmental research database. All patients fulfilled consensus diagnostic criteria for the relevant syndromic diagnosis (62–64) and all had clinically mild to moderate severity disease. Genetic screening revealed pathogenic mutations in twenty-two cases (eight *C9orf72*, all bvFTD; seven *MAPT*, 6 bvFTD and 1 SD; seven *GRN*, 4 bvFTD and 3 PNFA). Brain MRI was consistent with the syndromic diagnosis in all patients and none had evidence of significant cerebrovascular burden. Cerebrospinal fluid profiles of tau and beta-amyloid_1−42_ were available for twelve patients with typical AD and six patients with LPA; each was consistent with underlying Alzheimer's pathology, based on local reference ranges (total tau/beta-amyloid_1−42_ ratio >0.8). In all patients, the syndromic diagnosis was further corroborated by a comprehensive general neuropsychological assessment. We recorded the use of medications (antidepressants and neuroleptics) that could potentially affect time perception among the participant groups (65, 66). Clinical, demographic, and neuropsychological characteristics of all participant groups are summarized in Table 1.

**Table 1:** General demographic, clinical and neuropsychological characteristics of participant groups.

**Characteristics**	**Controls**	**bvFTD**	**SD**	**PNFA**	**LPA**	**AD**
**General demographic and clinical**						
No. (M/F)	32 (16/16)	34 (26/8)	17 (10/7)	20 (10/10)	9 (8/1)	28 (13/15)
Age (y)	68.2 (6.9)	65.8 (6.9)	66.5 (7.5)	68.5 (8.4)	69.2 (9.6)	70.4 (7.8)
Handedness (R/L)	29/2	32/1	17/0	18/1	8/1	25/2
Education (y)	16.1 (2.4)	13.8 (4.0)	15.0 (2.9)	13.6 (2.5)	16.2 (2.1)	14.9 (2.0)
MMSE (/30)	29.8 (0.4)	22.4 (6.4)	21.8 (8.0)	18.4 (9.5)	13.1 (7.8)	18.1 (6.6)
Symptom dur (y)	N/A	7.2 (5.0)	6.1 (2.4)	4.3 (2.4)	5.2 (1.9)	6.9 (3.6)
Medication use^**^: no (%)	2 (0.6)	16 (47)	6 (35)	8 (40)	2 (22)	13 (46)
**Neuropsychological**						
***General intellect***						
WASI VIQ	123.7 (8.2)^a^	**82.4 (27.6)^c^**	**67.9 (18.3)^a^**	**72.3 (18.9)^b^**	**61.3 (19.1)**	**93.1 (20.2)^a^**
WASI PIQ	125.2 (12.9)^a^	**92.8 (22.9)^c^**	114.5 (17.5)^a^	**88.8 (22.4)^b^**	**81.7 (12.9)**	**82.6 (16.7)^a^**
***Episodic memory***						
RMT Words (/50)	48.8 (1.2)^a^	**28.4 (18.8)^g^**	**26.3 (16.3)^d^**	**31.1 (18.4)^b^**	**20.2 (20.3)**	**25.4 (11.5)^e^**
RMT Words (/25)^*^	24.7 (0.8)	N/A	N/A	N/A	N/A	**15.3 (3.5)**
RMT Faces (/50)	43.9 (5.0)^a^	**23.7 (15.8)^f^**	**26.9 (11.7)^b^**	**30.5 (15.8)^b^**	**19.9 (19.7)**	**26.7 (11.7)^e^**
RMT Faces (/25)^*^	24.6 (0.7)	N/A	N/A	N/A	N/A	**17.8 (2.8)**
***Executive function***						
DS-F (max)	7.2 (1.1)^a^	**5.7 (1.4)^a^**	6.6 (0.9)^a^	**3.8 (2.2)^c^**	**3.0 (2.4)**	**5.9 (1.4)^a^**
DS-R (max)	5.5 (1.3)^a^	**3.6 (1.8)^a^**	5.0 (1.5)^a^	**1.9 (1.6)^c^**	**1.8 (1.4)**	**3.3 (1.8)^b^**
D-KEFS Stroop:						
Color (s)	30.1 (4.9)^b^	**53.8 (22.0)^a^**	**52.6 (23.2)^a^**	**77.3 (19.8)^d^**	**81.9 (13.7)**	**57.4 (17.1)^d^**
Word (s)	23.3 (5.0)^b^	**35.5 (19.0)^a^**	32.0 (18.4)^a^	**70.3 (25.5)^d^**	**60.8 (22.8)**	**44.4 (22.5)^d^**
Interference (s)	54.8 (13.2)^b^	**119.9 (54.8)^a^**	**96.9 (45.9)^a^**	**155.9 (44.8)^d^**	**180.0 (0.0)**	**145.6 (40.5)^d^**
Fluency:						
Verbal (total)	17.7 (5.7)^a^	**6.6 (5.4)^a^**	**6.9 (5.4)^a^**	**4.1 (4.4)^d^**	**2.0 (2.9)**	**8.9 (4.9)^b^**
Category (total)	24.6 (5.4)^a^	**10.0 (6.9)^a^**	**5.3 (4.4)^a^**	**9.4 (7.2)^d^**	**2.2 (2.9)**	**8.0 (5.0)^a^**
TMT A (s)	31.1 (9.2)^a^	**75.7 (46.4)^a^**	53.5 (27.7)^a^	**82.1 (45.4)^d^**	**116.9 (37.6)**	**99.3 (42.4)^a^**
TMT B (s)	60.2 (24.1)^a^	**202.2 (93.5)^a^**	**147.3 (88.4)^a^**	**229.2 (94.4)^c^**	**300.0 (0.0)**	**269.7 (69.0)^c^**
***Language skills***						
BPVS (/150)	148.0 (1.4)^a^	**110.7 (45.5)^e^**	**62.1 (39.8)^b^**	**117.6 (44.4)^b^**	**92.6 (55.0)**	**124.1 (36.7)^a^**
GNT (/30)	26.6 (2.7)^a^	**12.1 (9.3)^d^**	**1.0 (4.0)^a^**	**10.7 (7.2)^c^**	**7.0 (8.5)^a^**	**12.5 (8.3)^b^**
***Other skills***						
VOSP (/20)	18.9 (1.2)^a^	**14.2 (5.2)^d^**	**14.1 (4.7)^a^**	**15.1 (4.7)^b^**	**13.6 (3.7)**	**15.4 (2.6)^a^**

*Mean (standard deviation) values are shown unless otherwise indicated (maximum scores on neuropsychological tests are in parentheses); significant differences in performance between healthy controls and patient groups (p < 0.05) are coded in bold*.

* *based on data from an historical cohort of 24 healthy older controls and six patients with AD from the present cohort*;

** *includes medications with a potentially relevant effect on time perception (see text)*.

*A reduced number of participants completed certain tests, as follows*:

a *n-1*,

b *n-2*,

c *n-3*,

d *n-4*,

e *n-7*,

f *n-9*,

g *n-11*.

*AD, patient group with typical Alzheimer's disease; BPVS, British Picture Vocabulary Scale (67); bvFTD, patient group with behavioral variant frontotemporal dementia; Controls, healthy control group; D-KEFS, Delis Kaplan Executive System (68); dur, duration; F, female; GDA, Graded Difficulty Arithmetic test (69); GNT, Graded Naming Test (70); LPA, patient group with logopenic aphasia; M, male; MMSE, Mini-Mental State Examination score (71); N/A, not applicable; PIQ, performance IQ; PNFA, patient group with progressive non-fluent aphasia; RMT, Recognition Memory Test (72); SD, patient group with semantic dementia; TMT, Trails Making Test (73); VIQ, verbal IQ; VOSP, Visual Object and Space Perception Battery – Object Decision test (74); WASI, Wechsler Abbreviated Scale of Intelligence (75); DS-F/R, Wechsler Memory Scale (Revised) Digit Span – Forward/Reverse (76); y, years*.

All participants gave informed consent for their involvement in the study. Ethical approval was granted by the University College London and National Hospital for Neurology and Neurosurgery Joint Research Ethics Committees in accordance with Declaration of Helsinki.

### Assessment of Temporal Awareness

We surveyed the presence of behavioral symptoms suggesting an alteration of subjective temporal awareness (Table 2). We sampled temporal behavioral symptoms that we felt were likely to be pertinent based on our accumulated clinical experience of the target syndromes. These symptoms comprised: apparent confusion about the temporal ordering of experienced past personal events and/or how long ago such events occurred or will occur in future (i.e., difficulty estimating the interval separating the present from the past/prospective event); reduced temporal flexibility (temporal rigidity, exemplified by high valuation of punctuality, and discomfort if schedules were disturbed) and/or clockwatching (looking at their watch or asking for the time very often); and an increased tendency to re-live personal events from the past (as indicated, for example, by a conversational preoccupation with such events). The survey was completed by healthy controls and by each patient's primary caregiver; involvement of caregivers (either the patient's spouse or child) was intended to maximize understanding, communication, and accuracy of symptom reporting, since people with dementia (particularly FTD syndromes) often have limited insight into their own illness. For each of the sampled symptoms of altered temporal awareness, survey respondents were asked to indicate whether or not prominent changes (i.e., evident most days) had occurred. Caregivers were asked to compare patients' current behavior with their behavior premorbidly while healthy controls were asked whether they felt there had been any changes in their own behavior, referenced in each case to the situation 10 years previously: this interval reflects the typical duration of clinical symptoms in the target diseases plus some allowance for any prodromal changes. In addition, respondents were given the opportunity to make free comments, to provide further details about temporal behavioral alterations.

**Table 2:** Survey used to identify alterations in temporal awareness.

**Temporal symptom**	**Questions**
	*Thinking about [her / his / your] activities most days, please indicate whether or not you feel there has been a clear increase in any of the following*
Ordering past events	Confusion about the order in which personal events have happened
Estimating intervals between events	Difficulty estimating how long ago personal events occurred/how far in the future events will occur
Temporal rigidity	Intolerant of delays, anxiety or irritation about missing appointments or late arrivals, insistence on doing things at a particular time
Clockwatching	Tendency to “watch the clock” or preoccupation with the time
Re-living past events	Tendency to re-live personal events or episodes from the past

*Survey symptom items were chosen based on clinical observations of target disease groups and informed by previous studies of temporal awareness in the healthy brain (see text). The questionnaire was completed by healthy control participants themselves and by patients' primary caregivers. Respondents were asked to indicate whether or not prominent changes (i.e., evident most days) had occurred, for each of the sampled symptoms of temporal awareness. Caregivers were asked to decide whether changes had occurred comparing patients' current behavior with their behavior when well; healthy controls were asked to decide if changes had occurred in their own behavior over the past 10 years*.

### Analysis of Clinical and Behavioral Data

Clinical and behavioral data were analyzed using Stata version 14.0 software (StataCorp, College Station, TX, USA). Participant groups were compared using a one-way ANOVA for continuous variables satisfying normality criteria, or the non-parametric equivalent Kruskal-Wallis test if this was not the case. *Post-hoc* tests of non-parametric continuous variables were performed using Dunn's test. For categorical variables, we used the chi-square test, or Fisher's exact test when expected counts were small.

Survey data were analyzed to determine the prevalence of changes in temporal awareness for each participant group. Temporal awareness symptoms were coded as 1 (present) or 0 (absent). Because for three of the five symptom items no healthy control participant exhibited the symptom, we did not build a logistic regression model to compare the prevalence of alterations on that item for all patient groups vs. the healthy control group. Instead, we performed a two-tailed Fisher's exact test for each item and corrected for multiple comparisons using the Benjamini-Yekutieli procedure (77). For each temporal awareness symptom, once we had established an overall disease effect for that symptom, we used a logistic regression model to compare the log odds of exhibiting that symptom (as the dependent variable) between patient groups. We specified a dummy variable for diagnosis (our main variable of interest) taking the AD group as the reference and included age, gender and Mini-Mental State Examination (MMSE) score as covariates, to take into account of potentially confounding effects from these factors. MMSE score here served as an index of overall disease severity; although there is no single, principled index of severity for FTD syndromes (and all candidate severity measures are to some extent problematic and potentially confounded by linguistic and other considerations), the MMSE is a simple, widely used index that can be applied across participant groups. We also built logistic regression models to assess possible correlations between temporal awareness symptoms. Finally, we looked for any associations between altered temporal awareness and general clinical covariates (age, gender, years of education, MMSE score, relevant medication use) using the Student's *t*-test or the Wilcoxon rank sum test for continuous variables, and the chi-square test for categorical variables. A statistical significance threshold *p* < 0.05 was accepted for all tests.

### Brain Image Acquisition and Analysis

Volumetric brain MRI data from 91 patients were entered into the VBM analysis; scans were unavailable for 14 patients (three bvFTD, one PNFA, two LPA, eight AD) and a further three (two bvFTD, one PNFA) were inadequate on technical grounds. For each patient, a sagittal 3D magnetization-prepared rapid-gradient echo T1-weighted volumetric brain MR sequence (TE/TR/TI 2.9/2200/900 ms, dimensions 256 × 256 × 208, voxel volume of 1.1 × 1.1 × 1.1 mm) was acquired on a Siemens Prisma 3T MRI scanner using a 32-channel phased array head-coil. Pre-processing of brain images was performed using the New Segment and Diffeomorphic Anatomical Registration Through Exponentiated Lie Algebra (DARTEL) toolboxes in SPM12 (www.fil.ion.ucl.ac.uk/spm/software/spm12/), following an optimized protocol (78). Normalization, segmentation, and modulation of gray and white matter images were carried out using default parameter settings. Gray matter images were subsequently smoothed using a 6 mm full width-at-half-maximum Gaussian kernel. For each patient, total intracranial volume was calculated by combining gray matter, white matter, cerebrospinal fluid volumes after segmentation of these tissue types. A study-specific template brain image was created by warping all native space whole-brain images to the final DARTEL template and calculating the average of the warped brain images.

Across the combined patient cohort, we ran a full factorial model to assess associations of regional gray matter volume with each temporal symptom. The model incorporated the five temporal symptom items (coded as 0/1 for absence/presence of that symptom, respectively), diagnosis as a five-level factor, as well as age, total intracranial volume, and MMSE score as nuisance covariates. Negative (inverse) associations with regional gray matter (i.e., associations with gray matter atrophy) were assessed for every symptom item; positive gray matter associations were additionally assessed for symptoms of temporal rigidity, clockwatching, and re-living the past, since these phenomena are likely *a priori* to require at least partially preserved temporal processing mechanisms (58). Statistical parametric maps were generated using an initial threshold *p* < 0.001 and evaluated at peak voxel statistical significance level *p* < 0.05, after family-wise error (FWE) correction for multiple voxel-wise comparisons, separately within individual pre-specified neuroanatomical regions of interest. These regions were selected *a priori* based on functional neuroanatomical substrates of subjective time awareness identified in the healthy brain comprising anterior temporal lobe (the anterior parts of the superior, middle, inferior temporal, and fusiform gyri, and the temporal pole) (27, 79–81), insular cortex (2, 5, 13, 82), parietal cortex (inferior and superior parietal lobules, precuneus, and posterior cingulate cortex) (14–17, 83) and hippocampus (3, 19, 20). Regions were defined for the right and left hemispheres using the Harvard-Oxford Brain Atlas (http://fsl.fmrib.ox.ax.uk/fsl/fslwiki/Atlases). The regions used are presented in Figure S1.

## Results

### General Clinical and Neuropsychological Data

Participant groups (see Table 1) did not differ in age [*F*(5,134) = 1.31; *p* = 0.2619], nor handedness (*p* = 0.885); but differed significantly in gender balance [X^2^(5,140) = 11.204; *p* = 0.047] and years of education [X^2^(5,138) = 15.212; *p* = 0.0095]; the absolute difference was small, of the order of two years). Syndromic groups did not differ significantly in clinical illness duration [X^2^(4,103) = 8.43; *p* = 0.077]. However, there was a significant difference in MMSE scores [*F* (4,103) = 3.66; *p* = 0.0078], attributable in a *post hoc* analysis to significantly higher scores in the bvFTD group compared with the LPA group (*p* = 0.012).

### Temporal Awareness Symptom Data

Data on the prevalence of temporal awareness alterations for all participant groups are summarized in Table 3. Examples of caregiver comments about patients' behavioral alterations are provided in Table S1. The logistic regression analysis comparing syndromic groups is presented in Table 4. Logistic regression analysis probing temporal symptom correlations are also presented in Table S2. Prevalence data for altered temporal awareness in individual patients with genetic mutations causing FTD are summarized in Table S3.

**Table 3:** Proportions of participant groups with altered time awareness.

**Temporal symptom**	**Controls**	**bvFTD**	**SD**	**PNFA**	**LPA**	**AD**
	***n* = 32**	***n* = 34**	***n* = 17**	***n* = 20**	***n* = 9**	***n* = 28**
Ordering past events	0%	62%	12%	15%	56%	68%
Estimating intervals between events	0%	59%	18%	35%	67%	79%
Temporal rigidity	0%	41%	65%	35%	11%	11%
Clockwatching	3%	44%	59%	35%	22%	18%
Re-living past events	9%	59%	47%	25%	22%	21%

*Data derived from the customized questionnaire (see text and Table 2) were used to calculate the prevalence of time awareness alterations in each participant group, summarized here as proportion (percentage) of the group showing an alteration (total group sizes shown above). All patient groups combined were significantly different from the healthy control group (p < 0.001 after correction for multiple comparison)*.

**Table 4:** Results of the logistic regression analysis over the patient cohort.

**Temporal symptom**	**Variable**	**OR**	**95% CI**	***P*-value**
Ordering past events	Diagnosis			
	*bvFTD*	0.94	0.28–3.12	0.926
	*SD*	0.06	0.01–0.35	**0.002**
	*PNFA*	0.06	0.01–0.30	**0.001**
	*LPA*	0.34	0.06–1.91	0.219
	Gender (F)	0.91	0.33–2.50	0.852
	Age	0.97	0.92–1.04	0.407
	MMSE	0.92	0.86–0.98	**0.010**
	Constant	71.55	0.77–6633.90	0.065
Estimating intervals between events	Diagnosis			
	*bvFTD*	0.51	0.14–1.82	0.302
	*SD*	0.06	0.01–0.31	**0.001**
	*PNFA*	0.12	0.03–0.49	**0.003**
	*LPA*	0.33	0.05–2.02	0.232
	Gender (F)	1.03	0.39–2.71	0.948
	Age	0.99	0.93–1.05	0.666
	MMSE	0.91	0.86–0.97	**0.005**
	Constant	51.48	0.62–4292.08	0.081
Temporal rigidity	Diagnosis			
	*bvFTD*	5.50	1.24–24.40	**0.025**
	*SD*	17.33	3.30–91.09	**0.001**
	*PNFA*	5.29	1.11–25.14	**0.036**
	*LPA*	1.04	0.09–12.41	0.975
	Gender (F)	0.52	0.19–1.41	0.197
	Age	1.05	0.99–1.11	0.132
	MMSE	1.04	0.98–1.11	0.203
	Constant	0.00	0.00–0.29	**0.013**
Clockwatching	Diagnosis			
	*bvFTD*	4.44	1.21–16.35	**0.025**
	*SD*	8.98	2.09–38.58	**0.003**
	*PNFA*	2.80	0.70–11.12	0.146
	*LPA*	0.90	0.13–6.41	0.919
	Gender (F)	0.48	0.18–1.24	0.130
	Age	1.06	1.00–1.12	0.054
	MMSE	10.98	0.92–1.04	0.450
	Constant	0.01	0.00–0.57	**0.026**
Re-living past events	Diagnosis			
	*bvFTD*	3.49	1.05–11.63	**0.042**
	*SD*	2.40	0.61–9.47	0.212
	*PNFA*	1.12	0.28–4.47	0.875
	*LPA*	1.06	0.16–6.93	0.951
	Gender (F)	0.66	0.26–1.66	0.378
	Age	0.97	0.91–1.02	0.256
	MMSE	1.04	0.98–1.11	0.154
	Constant	1.56	0.03–96.61	0.832

*The Alzheimer's disease group is the reference for comparisons between diagnostic groups; significant associations with particular variables (p < 0.05) are coded in bold. bvFTD, patient group with behavioral variant frontotemporal dementia; CI, confidence interval; F, female; LPA, patient group with logopenic progressive aphasia; MMSE, Mini-Mental State Examination score; OR, odds ratio; PNFA, patient group with progressive non-fluent aphasia; SD, patient group with semantic dementia*.

Raw prevalence data (Table 3) indicated that alterations of temporal awareness were frequent in all syndromic groups but experienced by only a small minority of healthy controls. Overall, patients with probable AD pathology (AD and LPA) most frequently exhibited confusion ordering past events or difficulty estimating intervals between events; while patients with FTD pathology (bvFTD, SD, PNFA) were most frequently prone to temporal rigidity, clockwatching and/or a tendency to re-live past events. Certain temporal symptoms were especially salient in particular syndromic groups (present in over half the cases in that group): event ordering confusion and/or difficulty estimating intervals between events in bvFTD, LPA, and AD; temporal rigidity and clockwatching in SD; and a tendency to re-live past events in bvFTD. Difficulties with interval estimation was significantly correlated with temporal rigidity, whereas no significant associations were found between these symptoms and a tendency to re-live past events (Table S2).

Compared to healthy controls, the patient cohort overall had a significantly higher prevalence of confusion about ordering events in time and difficulty estimating intervals between events (both *p* < 0.0001). In the logistic regression analysis comparing syndromic groups, there was a main effect of diagnosis for both confusion of temporal order [X^2^(4, 103) = 20.70, *p* = 0.0004] and interval estimation difficulties [X^2^(4, 103) = 16.29, *p* = 0.0026]. Such disturbances were significantly more prevalent in the AD group than the SD group [temporal ordering: odds ratio (OR) = 0.06, 95% confidence interval (CI) 0.01–0.35; temporal estimation: OR = 0.06, CI 0.01–0.31] and the PNFA group (temporal ordering: OR = 0.06, CI 0.01–0.30; temporal estimation: OR = 0.12, CI 0.03–0.49).

Compared to healthy controls, the patient cohort overall also had a significantly higher prevalence of increased temporal rigidity and clockwatching (both *p* < 0.001). There was a main effect of diagnosis for clockwatching [X^2^(4, 103) = 10.57, *p* = 0.0318] and temporal rigidity [X^2^(4, 103) = 12.98, *p* = 0.0114]. Clockwatching was significantly more prevalent in the bvFTD and SD groups than the AD group (bvFTD: OR = 4.44, CI 1.21–16.35; SD: OR = 8.98, CI 2.09–38.58). Temporal rigidity was significant more prevalent in the bvFTD, SD, and PNFA groups than the AD group (bvFTD: OR = 5.50, CI 1.24–24.40; SD: OR = 17.33, CI 3.30–91.09; PNFA: OR = 5.29, CI 1.11–25.14).

Compared to healthy controls, patient groups overall were significantly more likely to re-live past events (*p* < 0.001). However, while this symptom was more prevalent in the bvFTD and SD groups, the logistic regression analysis showed no significant main effect of diagnosis [X^2^(4,108) = 5.78, *p* = 0.22], precluding further comparisons between disease groups.

Across the patient cohort, symptoms of disturbed past event ordering, or interval estimation were significantly associated with MMSE score (OR = 0.92, CI 0.86–0.98 and OR = 0.91, 95% CI 0.86–0.97, respectively). No other significant associations between developing temporal awareness symptoms and general patient characteristics (gender, age, education, or relevant medication use) were identified.

Considering the small subgroup of patients with genetic mutations (Table S3), symptoms of altered time awareness were generally frequent with all major mutations causing FTD. However, temporal rigidity was particularly associated with *MAPT* mutations, contrasting with its low prevalence in association with *C9orf72* and *GRN* mutations; no patients with *GRN* mutations were reported to have exhibited disturbances of temporal event ordering or interval estimation.

### Neuroanatomical Associations of Altered Time Awareness

**Figure 1:**
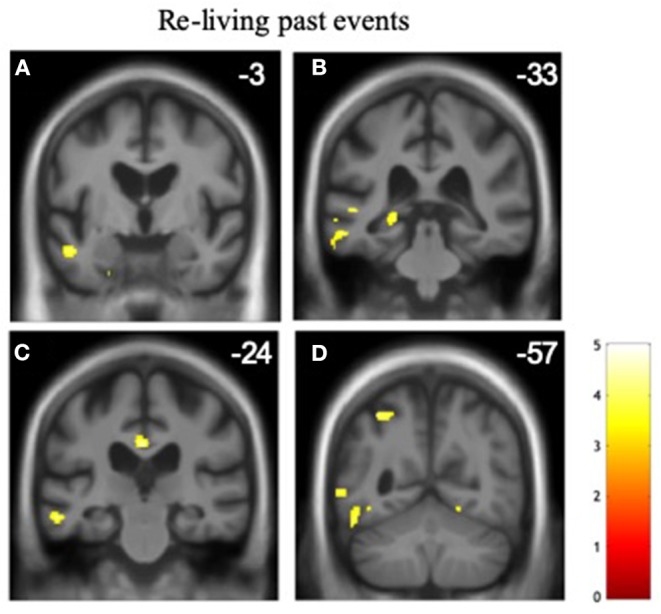
Gray matter associations of altered temporal awareness in the patient cohort. Statistical parametric maps show regional gray matter volume positively associated with the propensity to re-live past events across all syndromic groups (see text and Tables 2, 3 for details). For display purposes, maps have been rendered on coronal sections of the group mean template T1-weighted MR brain image and thresholded at *p* < 0.001, uncorrected for multiple voxel-wise comparisons over the whole brain volume (areas significant at p_FWE_ < 0.05 after correction for multiple comparisons within pre-specified neuroanatomical regions of interest are summarized in Table 5); the y-coordinate (mm) in MNI space of the plane of each section is indicated. The color bar codes voxel-wise T scores. The left hemisphere is shown on the left in all sections. The sections traverse the following key structures: **(A)** anterior middle temporal gyrus/superior temporal sulcus. **(B)** posterior hippocampus. **(C)** posterior cingulate cortex. **(D)** superior parietal lobule.

Significant gray matter associations of altered time awareness across the patient cohort are summarized in Table 5, all thresholded at p_FWE_ < 0.05 within pre-specified anatomical regions of interest; statistical parametric maps are presented in Figure 1. Across the combined patient cohort, increased tendency to re-live past events was associated with relatively preserved gray matter in a distributed left-sided network including anterior middle temporal gyrus and superior temporal sulcus, hippocampus, posterior cingulate, and superior parietal cortices. No other neuroanatomical associations of altered temporal awareness were identified.

**Table 5:** Neuroanatomical associations of altered time awareness in the patient cohort.

**Region**	**Side**	**Cluster (voxels)**	**Peak (mm)**			**T score**	**P_**FWE**_**
			**x**	**y**	**z**		
Middle temporal gyrus/superior temporal sulcus	L	118	−50	−3	−26	3.95	0.038
Hippocampus	L	31	−22	−33	−4	3.74	0.019
Posterior cingulate	L	184	−2	−24	33	4.52	0.015
Superior parietal lobule	L	75	−32	−57	51	4.23	0.038

*The table presents the locations of regional gray matter positively correlated with tendency to re-live past events (the only significant association of altered time awareness identified) over the combined patient cohort, based on voxel-based morphometry (see text and Tables 2, 3 for further details). Coordinates of local maxima are in standard MNI space. P-values were all significant (p < 0.05) after family-wise error (FWE) correction for multiple voxel-wise comparisons within pre-specified anatomical regions of interest (see text)*.

## Discussion

Here we have demonstrated alterations of multiple dimensions of long-duration, subjective time awareness in canonical syndromes of FTD, referenced to syndromes of AD as well as healthy older individuals. Abnormalities of time awareness were exhibited by over half of patients with bvFTD and SD as well as typical amnestic AD and LPA, and by around a third of patients with PNFA. However, the pattern of abnormalities was not uniform over the patient cohort: syndromic groups showed separable (albeit overlapping) profiles of altered time awareness, in line with our prior clinical hypotheses. Patients with typical AD and LPA had particularly salient difficulties with ordering past events and placing events in time, without significant changes in other aspects of temporal behavior sampled here; the pattern of temporal symptoms was much more heterogeneous in the FTD cohort. The SD group had prominent temporal rigidity and clockwatching, while the bvFTD group exhibited predominant abnormalities of past event ordering and re-living past events. Profiles of altered temporal awareness differed significantly between AD and FTD disease groups when directly compared and although there was an overall correlation of abnormal past event ordering and interval estimation with worsening disease severity, syndromic profiles of altered temporal awareness were evident after taking background clinical characteristics (age, gender, MMSE score) into account.

Informed by previous work in the healthy brain (84–87), we propose a tentative synthesis of these findings in terms of different “domains” of temporal awareness. Symptoms of abnormal event ordering or interval estimation might both plausibly reflect a disturbed mental timeline, while temporal rigidity and clockwatching reflect related aspects of mental timekeeping. The latter might be further related to the “Godot syndrome”, a phenomenon previously described in AD, and which refers to anxiety surrounding upcoming events (88, 89). Our findings corroborate previous reports of obsessional clockwatching in SD and bvFTD (31, 58) as well as time estimation difficulties in AD (46) but further illustrate that alterations of temporal awareness transcend canonical syndromic boundaries. Across syndromic groups, patients presenting with temporal rigidity, or clockwatching were less likely to also exhibit difficulty ordering past events and vice versa, but no association was found between those symptoms and a tendency to re-live past events. This is in line with the hypothesis that the propensity to re-live past events might constitute a partly compensatory phenomenon in the face of impoverished mental timeline, somewhat analogous to the normal phenomenon of “nostalgia” (77, 90).

Across the combined patient cohort, a tendency to re-live past events was associated with relative preservation of a distributed left-sided temporal, parietal, and hippocampal network. This is in line with an emerging picture of temporal processing and subjective temporal awareness derived from studies of the healthy brain. In particular, the parietal cortex has been implicated in reconstructing sequences of spatio-temporal events and their temporal ordering (15, 16, 91, 92). In conjunction with hippocampus as well as insular and prefrontal cortices, the parietal lobes participate in a distributed neural network that accesses and manipulates the mental timeline according to salience and behavioral context (82, 93, 94). This network further overlaps core elements of the default mode network which integrates information about current bodily states and memories with incoming sensory traffic. Moreover, the hippocampus is critical for initial encoding of events and their embedding in emotional context (3, 19, 20, 95), and the anterior temporal lobe is likely to be important for the semantic integration of autobiographical events (96). The integrity (or partial integrity) of these brain areas might plausibly support a tendency to re-live past autobiographical events, in line with previous evidence in neurodegenerative syndromes (97–99).

The lack of gray matter associations of other symptoms of altered temporal awareness here is, at first sight, a little surprising. However, there are several factors that might potentially account for these results. Firstly, the neurobiological status of the temporal awareness “symptoms” we considered here are different between symptom categories. Re-living of past events depends to some extent on a neuroanatomical substrate that is at least partly preserved (since temporal events have to be represented and accessed in order to be re-lived). It is therefore plausible that this substrate should be identified across syndromic groups, since it reflects the architecture of the healthy brain. On the other hand, we sampled other temporal symptoms that directly reflect the impact of neurodegenerative brain damage, which will have varied between syndromes and is therefore less likely to have a common neuroanatomical association across the patient cohort. In addition, it is possible that the neuroanatomical substrates of these other temporal symptoms are extensively distributed, widely variable between individuals or alternatively, highly convergent between symptom categories: any of these scenarios would have made neuroanatomical associations less liable to be identified using the VBM model we employed here. It is further plausible that at least some symptoms may arise from network connectivity changes that are not captured using VBM. Finally, the binarised symptom classification here may well have reduced scope to detect gray matter associations that might have been evident with a continuously distributed variable (e.g., a symptom severity score). It should be kept in mind that these factors are, to some extent, limitations imposed by the VBM technique itself (which is essentially a correlational methodology) rather than specific to temporal processing per se.

Furthermore, cellular and molecular as well as macro-anatomical factors are likely to influence temporal processing and subjective temporal awareness in neurodegenerative pathologies (100). The small case numbers here preclude firm conclusions concerning the temporal awareness profiles of particular genetic mutations. However, it is noteworthy that temporal rigidity and clockwatching were not reported in patients with *GRN* mutations, whereas these were salient symptoms in patients with *MAPT* mutations. It may be relevant that *MAPT* mutations target the antero-mesial temporal lobes relatively selectively (101) while *GRN* mutations frequently target parietal cortex (102). These issues will only be resolved by further neuroanatomical work addressing functional as well as structural network connectivity.

From a clinical perspective, our findings endorse the long-held bedside impression of temporal obsessionality in SD and bvFTD, while further corroborating reports of disordered temporal estimation in AD. Quantification of changes within the temporal symptom categories we have foregrounded here would help in planning, implementing and evaluating new behavioral interventions designed to help patients orient and navigate in time and to reduce patient and caregiver distress incurred by abnormal temporal reactivity. The overall correlation of mental timeline abnormalities with advancing disease noted here accords with previous suggestions that clockwatching behavior may be restricted to the earlier stages of SD (58). Our observations here could motivate further work to develop quantifiable cognitive tests of temporal awareness. Such tests might, in future studies, yield novel functional biomarkers that index the integrity of temporal processing mechanisms in neurodegenerative syndromes.

This study has several limitations that should inform future work. Most fundamentally, there is a need to further corroborate the results in larger patient cohorts and ideally with histopathological and molecular correlation. It would be of considerable interest to compare the profiles of altered temporal awareness exhibited by patients with FTD and AD directly with other neurodegenerative pathologies, for example Lewy body disease, which also target brain systems implicated in temporal perception. It will also be relevant to study these profiles longitudinally: the phenomenology of neurodegenerative syndromes is dynamic and multiphasic, while both in patients and healthy individuals, subjective temporal awareness may be modulated by key life events (such as retirement from work). The categories of temporal symptoms assessed in this first study were intentionally broad and qualitative, designed to capture a diverse range of phenomena. However, these symptom categories should be unpacked in further studies to quantify the frequency and severity of symptoms that patients experience and to capture the nature of their difficulties more precisely. For example, “difficulty” estimating the temporal intervals between events could mean simply that patients fail to take account of lapsed time or rather that they express unreasonable estimates of the relevant intervals. In turn, these processes might plausibly be affected differentially in different syndromes and diseases (in particular, AD vs. FTD). Particularly with regard to symptoms suggesting disturbances of the mental timeline, it will be crucial to define how such disturbances relate to deficits of episodic memory and the detail with which particular events and their spatio-temporal context are encoded. It will also be important to acquire patients' own reports of time awareness, ideally in parallel with caregivers' perspectives. Certain aspects of temporal awareness (for example, capacity to envisage the future) are intrinsically difficult to capture from second-person questionnaires but potentially highly illuminating in particular neurodegenerative syndromes [such as bvFTD and SD (53, 55)]. Our findings provide a prima *facie case* for the future design and validation of temporal symptom scales relevant to a broad range of neurodegenerative diseases.

Understanding the neural mechanisms of altered temporal awareness associated with these neurodegenerative pathologies will require a detailed assessment of temporal perception (in particular the psychophysical correlates of interval and pattern processing), consideration of accompanying behavioral phenotypes [since altered emotional reactivity is very likely to impact on temporal behaviors (7)], and functional neuroimaging techniques that can capture dynamic interactions and connectivity between brain network elements. In this regard, magnetoencephalography would be a particularly attractive modality, by virtue of its high temporal resolution and capacity to track changes in cortical laminar physiology. The last would offer the exciting prospect of relating complex temporal behavioral phenotypes to dysfunction at the level of tissue microcircuits and synapses, which is likely to be apposite in light of emerging evidence that neural mechanisms of temporal awareness span scales ranging from the cellular to the macroscopic (4, 5). Indeed, this is arguably a compelling motivation for developing true “temporal biomarkers,” since indices of universal brain processes (such as temporal processing) are likely to prevail across pathologies and disease stages.

This preliminary study calls attention to the significance of temporal awareness symptoms and pertinent neural network substrates across major dementia syndromes. The findings provide a rationale for a more systematic analysis of subjective time in neurodegenerative pathologies, with a view to developing validated clinical assessment tools, understanding underlying neurobiological mechanisms and designing management interventions.

## Data Availability Statement

The datasets used and/or analyzed during the present study are available from the corresponding author on reasonable request.

## Ethics Statement

The studies involving human participants were reviewed and approved by University College London and National Hospital for Neurology and Neurosurgery Joint Research Ethics Committees. The patients/participants provided their written informed consent to participate in this study.

## Author Contributions

CM and JW were responsible for the conception and design of the study. CM and HS carried out symptom survey data collection. LR, CG, KM, EB, and JR were responsible for recruitment, neuropsychology, and imaging data acquisition. M-CR-K was responsible for the analysis of the survey data and M-CR-K, CM, CH, RB, and JA for the voxel-based morphometry analysis. M-CR-K, CM, and JW were responsible for the interpretation of the data. M-CR-K and JW were responsible for drafting the manuscript. M-CR-K, CM, CH, and JW for revisiting it critically for important intellectual content. All authors read and approved the final manuscript.

## Conflict of Interest

The authors declare that the research was conducted in the absence of any commercial or financial relationships that could be construed as a potential conflict of interest.
