# Supplementary material for: Altered Time Awareness in Dementia
Source: Front Neurol. 2020 Apr 21;11:291. doi: 10.3389/fneur.2020.00291 (PMC7186333; doi:10.3389/fneur.2020.00291)
Supplement: Supplementary file 1 [file Table_1.DOCX]

**SUPPLEMENTARY MATERIAL**

**Altered time awareness in frontotemporal dementia, by M-C Requena-Komuro et al.**

| **Temporal symptom** | **Representative caregiver comments** | **Syndrome** |
| --- | --- | --- |
| Confusion ordering events / difficulty estimating intervals between events | No concept of the passage of time | bvFTD |
| Temporal rigidity | Needs to know exact times, very fixed with times (e.g., feeding the cat) | SD |
|  | Likes fixed mealtimes | PNFA |
|  | Likes fixed timing routines | AD |
| Clockwatching | Generally anxious about timing | bvFTD |
|  | Very anxious about the time | PNFA |
|  | Asks time often, transfixed with his watch | AD |
| Re-living past events | Often talking about university years, events from early life with parents | bvFTD |
|  | Very emotional about the past, especially recalling arguments | SD |

**Table S1.** Examples of caregiver responses to the temporal awareness questionnaire

AD, typical Alzheimer’s disease; bvFTD, behavioural variant frontotemporal dementia; PNFA, progressive nonfluent aphasia; SD, semantic dementia.

**Table S2.** Results of the logistic regression analysis comparing temporal symptoms across the patient cohort

| **Temporal symptom** | **Variable** | **OR** | **95% CI** | **P value** |
| --- | --- | --- | --- | --- |
| Difficulty estimating intervals between events | Temporal rigidity | 0.23 | 0.10-0.55 | **0.001** |
|  | Re-living past events | 1.08 | 0.47-2.48 | 0.851 |
|  | Constant | 1.83 | 1.04-3.22 | **0.037** |
| Temporal rigidity | Difficulty estimating intervals between events | 0.23 | 0.10-0.55 | **0.001** |
|  | Re-living past events | 1.30 | 0.55-3.10 | 0.555 |
|  | Constant | 0.91 | 0.47-1.73 | 0.763 |
| Re-living past events | Difficulty estimating intervals between events | 1.84 | 0.88-3.87 | 0.106 |
|  | Temporal rigidity | 2.05 | 0.91-4.61 | 0.082 |
|  | Constant | 0.29 | 0.15-0.80 | **< 0.001** |

Each time symptom was chosen in turn as the reference to assess correlations between symptoms in different time domains across the whole patient cohort; significant associations with particular variables (p<0.05) are coded in bold. CI, confidence interval; OR, odds ratio.

**Table S3.** Prevalence of altered time awareness in FTD patients with genetic mutations

| **Temporal symptom** | ***C9orf72*** | ***MAPT*** | ***GRN*** |
| --- | --- | --- | --- |
|  | n = 8 | n = 7 | n = 7 |
| Confusion ordering events | 63% | 57% | 57% |
| Difficulty estimating intervals between events | 63% | 71% | 57% |
| Temporal rigidity | 25% | 71% | 0% |
| Clockwatching | 50% | 71% | 0% |
| Re-living past events | 75% | 57% | 57% |

Mutation carriers are coded by site of mutation as follows: ***C9orf72*,** mutation in open reading frame 72 on chromosome 9; ***GRN***, progranulin; ***MAPT***, microtubule associated protein tau.

**Figure S1.** Anatomical regions of interest used in the VBM analysis


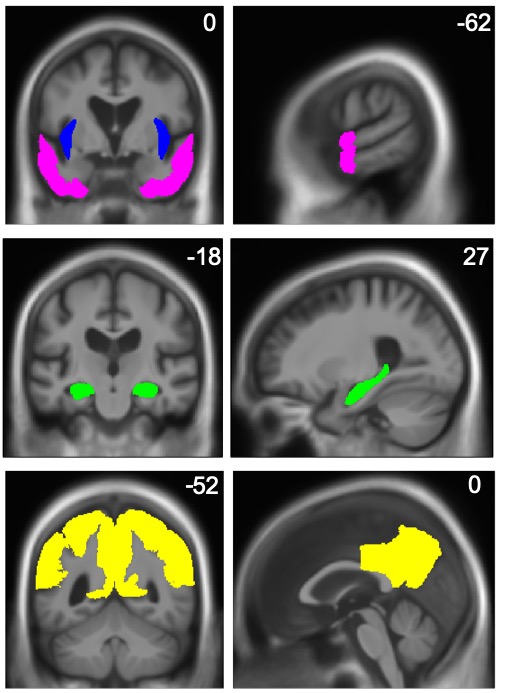


Coronal (left) and sagittal (right) views of the regions of interest used in the VBM analysis during small volume correction for multiple voxel-wise comparisons (see text for details). Regions have been overlaid on the group mean structural brain image in MNI space, coordinates (mm) of the plane of each section are indicated. Blue: insular cortex; magenta: anterior temporal lobe; green: hippocampus; yellow: parietal region (inferior and superior parietal lobules, precuneus, and posterior cingulate cortex).
